# A New Measure for Quantifying Four-Limb Coordination of Human Gait Based on Mobility Sensors

**DOI:** 10.3390/s24186105

**Published:** 2024-09-21

**Authors:** Noam Galor, Gabi Zeilig, Meir Plotnik

**Affiliations:** 1 Center of Advanced Technologies in Rehabilitation, Sheba Medical Center, Ramat Gan 5266202, Israel; noam.galor@sheba.health.gov.il; 2 Department of Neurological Rehabilitation, Sheba Medical Center, Ramat Gan 5266202, Israel; gabi.zeilig@sheba.health.gov.il; 3 Department of Physical and Rehabilitation Medicine, Faculty of Medicine, Tel Aviv University, Tel Aviv 6997801, Israel; 4 School of Health Professions, Ono Academic College, Kiryat Ono 5545001, Israel; 5 Department of Physiology and Pharmacology, Faculty of Medicine, Tel Aviv University, Tel Aviv 6997801, Israel; 6 Sagol School of Neuroscience, Tel Aviv University, Tel Aviv 6997801, Israel

**Keywords:** gait analysis, inter-limb coordination, rhythmic movements, locomotion, phase coordination index

## Abstract

Coordinated movement of four limbs is a hallmark of healthy locomotion. No measures exist to quantify four-limb coordination. This study aimed to investigate temporal four-limb coordination and proposed a new metric for quantifying the inter-limb phase of rhythmic locomotion-related movements. Kinetic data of arm and leg movements generated during walking (self-selected speed) from healthy adults were used to extract the phases (φ) between all possible limb pairings. The φ series were used to calculate each pair’s Phase Coordination Index (PCI). The PCI quantifies the accuracy and consistency of generating anti-phased rhythmic movements (lower PCI values mean better coordination). We also calculated the Quadruple-PCI (Q-PCI) by combining all φ values of all limb pairs. We found a significant correlation between the PCI values of all limb pairings and the Q-PCI (pairs involving arms: Pearson’s R > 0.79, *p* < 0.001; leg–leg: Pearson’s R = 0.3, *p* < 0.01). The PCI values that involve arms (median values between 6.5% and 8.3%) were significantly higher than the leg–leg PCI (median values between 3.8% and 4.1%), and the Q-PCI (median values between 8.3% and 9.7%) was significantly higher than all other PCI values. We also found a negative correlation between the arm swing amplitude and the PCI values (Spearman’s Rho of different limb pairings ranging from −0.25 to −0.5, *p* < 0.05), suggesting that higher arm swing amplitude leads to better coordination. Four-limb coordination analysis is a novel method for comprehensive assessment of gait coordination, which is often compromised among persons with disabilities.

## 1. Introduction

Human walking is characterized by highly coordinated rhythmic movements generated by all four limbs, with anti-phase coordination between the legs and arms. While the legs produce progression, the arms contribute to minimization of energy consumption [1,2,3,4,5] and help optimize physical stability [2,6,7,8,9]. Similar to the alternating motion of legs, the arms swing in a reciprocal manner. More specifically, arm swinging is often considered as a mechanism for counteracting free vertical moments (i.e., torque about the vertical axis) created by the legs, allowing forward progression by balancing the angular moments [10].

Human gait coordination of all four limbs has been studied [11,12], but still lacks a ‘metric’ for quantification. Since most neurological conditions associated with gait disorders involve deficits in both stepping and arm swinging movements [13,14,15], this quantification is important. Both rhythmic stepping and arm swinging movements performed by the relevant limbs involve synergistic activation of multiple muscles, each with a specific temporal activation pattern [16]. Muscle synergies are responsible for the coordinated movement of muscles by grouping them into functional units [17], i.e., various muscle synergies are associated with each stage of the gait cycle. It has been shown that the recruitment of muscle synergies at a particular stage explains the variation in locomotor patterns observed from one cycle to the next [18]. Therefore, the temporal coordination of walking involves intra-limb inter-muscular coordination of the walking person. Specifically, inter-limb coordination (e.g., anti-phase arm and leg movements) is relevant for minimizing walking cost [4] and increasing walking stability and performance [19].

From an anatomical perspective, the alternating rhythmic muscular activities of agonist and antagonist muscles acting on a joint during locomotion are (partially) controlled via central pattern generators (CPGs) [20]. This idea is supported by a large amount of evidence indicating the presence of spinal neuronal connections between the cervical and lumbar enlargements, likely where the CPGs are located. Thus, it is reasonable that the coordination between these two areas during locomotion is mediated by spinal pathways that receive sensory and supraspinal inputs. Animal studies have shown that the motor and sensory cortices monitor forelimb–hindlimb coordination and that synchronous activity can be observed in the cervical and lumbar ventral roots [21,22,23].

One of the open questions that remained unsolved is the directional influence of upper–lower limb coordination. Zehr and colleagues reviewed [21] the evidence about locomotion related forelimb–hindlimb coupling in animals, and upper–lower limb coupling in humans. The revealed picture is not cohesive. For example, in intact cats walking on a transverse split-belt treadmill, there is a differential effect of forelimb and hindlimb speed manipulation on forelimb–hindlimb coordination. When the forelimbs are forced to walk faster, a 2:1 fore–hind step relationship emerges. However, when the hindlimbs step faster, a 1:1 fore–hind step relationship is maintained [24,25]. In humans, on the other hand, an intended slowness of walking speed, achieved by a lower limb stepping pattern change, leads to a change in upper–lower cycle relation (from 1:1 to 2:1, [26,27]). Moreover, in order to support the existence of neural coupling between the upper and lower limbs during gait, Weersink and colleagues studied intermuscular coherence of gait-related electromyography from upper and lower limbs in healthy adults [28]. They identified significant coherence in several bands, suggesting that “*upper and lower limbs share common subcortical and cortical drivers that coordinate the rhythmic four-limb gait pattern*”. Based on their analyses, they also conclude “*that upper limb muscles drive and shape lower limb muscle activity during gait* via *subcortical and cortical pathways and to a lesser extent vice-versa*” [28], see also [29].

In humans, the assessment of inter-limb temporal coordination is commonly performed using mobility signals collected using mobility sensors, e.g., markers (i.e., of motion capture systems) placed on different parts of the body, or inertial measurement units (IMUs) such as accelerometers or gyroscopes, which are also attached to different parts of the body. The inter-limb coordination during locomotion in humans is primarily studied between bilateral homologous organs, i.e., the left leg in relation to the right leg, or the left arm in relation to the right arm. Ipsi-lateral and contra-lateral arm–leg coordination is less studied (but see [28,30]).

The aim of the current study was to assess coordination between each possible arm–leg pairing, including ipsi-lateral and contra-lateral pairs, and propose a novel measure to evaluate quadruple coordination of human gait. The newly proposed measure is based on the previously described Phase Coordination Index (PCI) [31], which quantifies the accuracy and consistency of the generation of left–right anti-phase movements (e.g., stepping of the legs; see more details on PCI calculations in *section* 2 below). The PCI has been shown to be sensitive to ageing [31,32] and to neurological conditions that affect gait, such as stroke [33,34], multiple sclerosis [35,36], and Parkinson’s disease [37,38]. We calculated the PCI for each possible pair of limbs by evaluating the phase between them. We then designated the new measure as Q-PCI (i.e., Q stands for quadruple); this measure assesses coordination of the four limbs during human locomotion. This report provides information on how to calculate the Q-PCI based on sensor data from all four limbs, and findings on the relationship between overall four-limb coordination (Q-PCI) and coordination between any possible limb pairings.

The main contributions of this study are:

- Providing an approach for assessing temporal coordination between all possible limb pairings (e.g., leg–arm) during walking.
- Developing a novel metric for quantifying quadruple coordination based on the accuracy and consistency of the interlimb phasing relation of the left–right stepping rhythmicity.
- Assessing the relationship between arm swing amplitude and inter limb coordination.

## 2. Materials and Methods

This study makes use of data collected from four experiments. Data were collected at the Center of Advanced Technologies in Rehabilitation in Sheba Medical Center. Table S1 in the Supplementary Materials, Section A, depicts the specific experimental protocols. All experiments were approved by the Sheba Medical Center institutional review board (IRB; ethical number 9407-12-SMC). All participants signed informed consent before enrolling in the study.

### 2.1. Participants

Data were collected from 71 healthy participants who completed a walking task (Table 1). Of these, 41 were young adults (YA; 21–40 years) and 30 were older adults (OA; 60–80 years). The demographic characteristics of the two groups are presented in Table 1. According to their self-reports the participants were in good health, and prior to the experiment it was verified that they could walk without the assistance of a walking aid.

### 2.2. Apparatus

**Figure 1:**
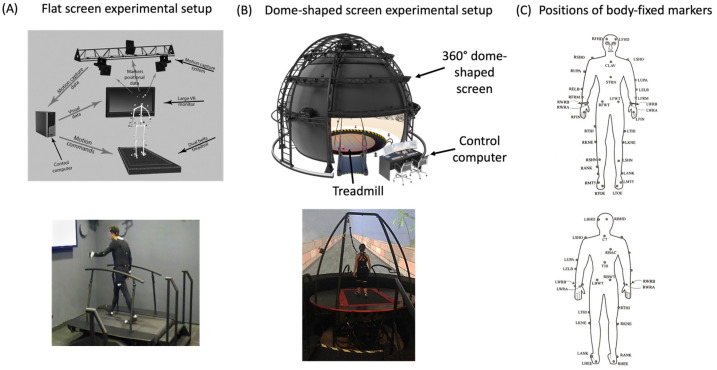
Experimental set up. (**A**) V-gait; (**B**) CAREN High END; (**C**) Marker positions.

The data used in the present study were collected using self-paced treadmills (TM), which are part of the following systems: (1) V-Gait (Motek Medical, the Netherlands), which includes a self-paced TM [39] (R-Mill, ForceLink, The Netherlands); or (2) Computer assisted rehabilitation environment (CAREN), CAREN High End (Motek Medical, The Netherlands). The latter is a fully immersive VR system (visual scenery is projected on 360° dome-shaped screen), and the former is a semi-immersive VR system (visual scenery is projected on a flat monitor screen). Figure 1 depicts the systems (panels A and B, respectively). All participants wore safety harnesses. An array of 41 passive markers was placed on each participant’s body (Figure 1C; segment model in use: ‘HumanRTKM’). A motion capture system (Vicon, Oxford, UK) recorded kinematic data using cameras (18 in the CAREN High End, 12 in the V-gait) that captured the location of the markers at a sampling rate of 120 Hz with a spatial resolution of 1 mm.

### 2.3. Procedure

**Figure 2:**
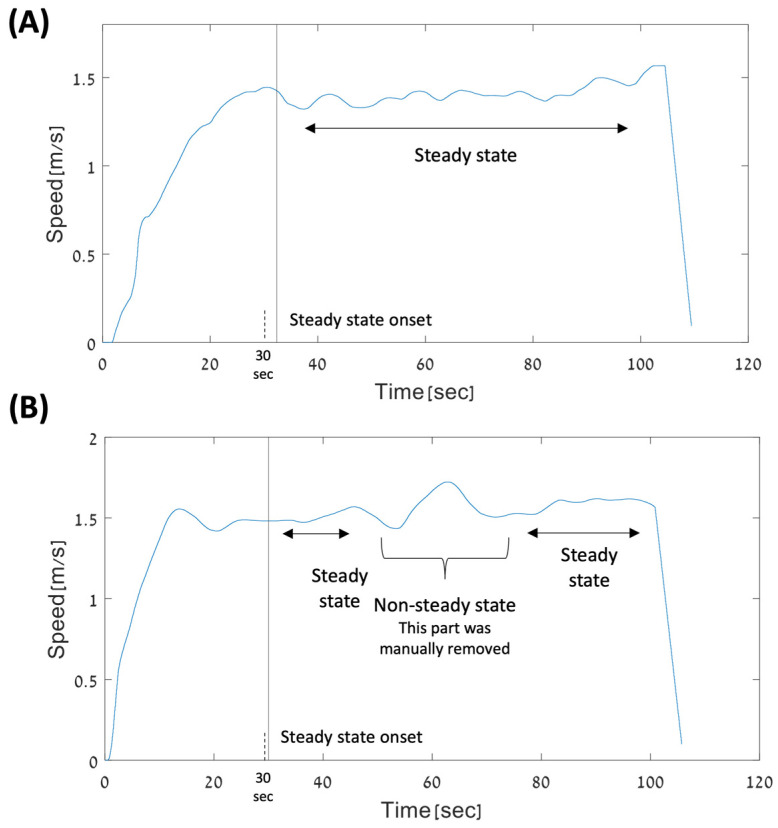
Selecting gait segments for the study. A black vertical line depicts the beginning of the analyzed walking segment. Top panel (**A**) presents a case where the participant maintained a stable walking speed starting at about 32 s after the experiment started. Lower panel (**B**) presents a case where the participant reached a steady state and then the gait speed became unstable (i.e., within a period of 20 s the speed rose and dropped again) and returned to steady-state walking. In this case, we did not include this 20 s segment or data from the two steady state segments (before and after the non-constant speed segment) were combined.

All four experimental paradigms whose data were included in the present study shared a walking period in steady state velocity in self-paced mode. Specifically, participants started from standing on the TM and were instructed to start walking at their self-selected comfortable pace. The TM speed automatically changed according to the participant walking speed. After a period of acceleration and acclimation, the participants reached a steady state (see Figure 2). From that point on, gait data were included in the analysis. Participants walked for a period of one to three minutes. A detailed description of the four experimental paradigms can be found in the Supplementary Materials Table S1.

### 2.4. Data Analysis

All analyses including data processing were performed using MATLAB (version R2021b, MathWorks, Natick, MA, USA) and Microsoft Excel (Microsoft Office 365, Microsoft, Redmond, Washington, DC, USA).

#### 2.4.1. Selecting Walking Periods

The start of the analyzed walking period was selected as the time after the participant reached a steady state velocity and after at least 30 s of walking (see Figure 2 for examples). We defined that a person reached the steady state when the coefficient of variance (CV) of gait speed was less than 2% (the CV was calculated in a 10 s moving window). Once the participant reached steady state, it was either maintained (i.e., the walking speed did not change and remained stable during the gait trial, Figure 2A) or non-stable (the walking speed changed (Figure 2B)). In this latter case, we manually removed the non-stable walking period from the analysis.

It was previously reported that during slow walking, the ratio between the arm swinging frequency and leg stepping is 2:1, while at a self-comfortable walking speed, this ratio was found to be 1:1 [26]. In the present study, we focused only on the latter case in order to study four-limb coordination during habitual walking (i.e., while the participants walked at their own comfortable speed). Therefore, in order to verify it was a comfortable speed, a verification of the ratio between the frequency of the steps and the arm swing was made. The mean ratio (±SD) across all participants (YA and OA) was 1.01 ± 0.06.

#### 2.4.2. PCI Definition and Calculation

The PCI is a measure that evaluates the coordination between two cyclic movements generated by different limbs. In walking, ideally, the step duration is half of the stride duration, meaning that if a full stride is defined as a cycle of 360°, the phase relationship between the left and right stepping is 180° (i.e., one step, half of one stride; or the heel strike (HS) of one leg, ideally, occurs in the mid duration between the consecutive heel strikes of the second leg).

In order to calculate the PCI, we first calculated the stepping phases (φ) between the right and the left leg for each gait cycle in the analyzed walking trial as shown in Equation (1):

(1) $$\varphi =360\frac{t_{Ai}-t_{Bi}}{t_{B\left(i+1\right)}-t_{Bi}}=360\frac{step\;time\;A}{Stride\;time\;B}$$

In this equation, $t_{Ai}$ and $t_{Bi}$ denote the time of the ith HS of one leg and the other leg, respectively (note that $t_{B(i+1)}$ > $t_{Ai}$ > $t_{Bi}$). φ was calculated relatively to gait cycles defined by the right leg (LreR stands for left leg stepping relative to right leg stepping), i.e., taking the timing of HS of the left leg relative to the duration between the preceding and the trailing heel strikes of the right leg. Separately, we repeated this calculation relative to gait cycles defined by the left leg (RreL stands for right leg stepping relative to left leg stepping).

The mean of all φ values obtained from the LreR analysis in a given gait segment corresponds to the mean of all φ values obtained from the RreL analysis. They both ‘reciprocate’ each other with respect to the ‘ideal’ 180° value. E.g., if mean $\varphi_{RreL}\;$= 184.1°, about 4° more than 180°, mean $\varphi_{LreR}$ = 176.1°, approximately 4° less than 180° (see Supplementary Materials, Section B, for more details).

After computing a vector of φ values relative to both sides (LreR or RreL, consistently for all gait cycles), the PCI was calculated as follows (Equation (2)):

(2) $$PCI\left[\%\right]=\varphi_{CV}+\varphi_{ABS}=100\frac{stdev(\varphi )}{mean(\varphi )}+100\frac{mean(\left|180-\varphi \right|)}{180}$$

where $\varphi_{CV}$ is calculated by the relation $100\frac{stdev(\varphi )}{mean(\varphi )}$ and reflects the consistency in the left–right stepping phase, and $\varphi_{ABS}$ is calculated by the relation $100\frac{mean(\left|180-\varphi \right|)}{180}$ and reflects the level of accuracy in generating the ideal 180° phasing. PCI was calculated both for the LreR and the RreL schemes.

The higher the PCI value, the less consistent (higher variability) and less accurate (the difference from 180° is bigger) the left–right phasing along gait cycles, i.e., gait is less coordinated [31].

**Figure 3:**
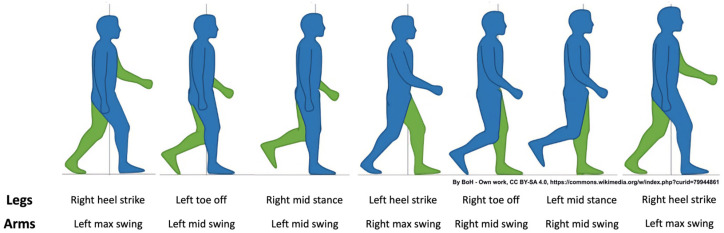
Gait cycle. Blue color represents right side (i.e., right leg and arm), green color represents left side (i.e., left leg and arm). The figure was adapted and modified from: BoH, CC BY-SA 4.0 <https://creativecommons.org/licenses/by-sa/4.0> (accessed on 5 May 2024), via Wikimedia Commons. Graphical modifications were made from the original, i.e., the human silhouettes were made tighter.

In this study, we generalized this approach for calculating PCI values for each possible pairing between the limbs. For this, we needed to define the relevant gait events in each limb’s activity. Using the timing of these events, we can calculate vectors of inter-limb φ values. Figure 3 illustrates the activity of all four limbs during the gait cycle (i.e., HS to HS of the same leg).

For the arms, the relevant events for defining phases with respect to other limbs is the time the rotation of the upper arm around the shoulder joint reaches its maximal angle with respect to the vertical axis, i.e., maximal forward swing (MFS; see Figure 3 right most silhouette for the left MFS event).

The following events were detected for each of the four limbs: right leg–right HS; left leg–left HS; right arm–MFS of the right arm; and left arm–MFS of the left arm. The timing of the heel strikes was derived from the vertical position of the markers located on the left and right heels; HS was defined as the point at which the marker reached a minimum position on the vertical axis. The timing and amplitude of MFS of the arms were derived from the spatial position of the markers located on the left and right elbows and shoulders as follows: we first calculated the shoulder angle vector (i.e., the arm swing amplitude vector), defined as the angle between the arm and the vertical axis, i.e., the angle between the vertical and anterior–posterior axes of the shoulder and elbow markers. Second, we extracted the timing of the MFS and the maximal backward swing (MBS) from the maximum and minimum points of the shoulder angle vector, respectively. The MFS amplitude of each gait cycle was defined as the difference between the angle at the MFS and at the MBS.

From the timing of the relevant events (i.e., HS and MFS), the PCI was calculated for all six possible limb pairings: (1) leg–leg for right and left legs; (2) arm–arm for right and left arms; (3) right arm and right leg (RaRl); (4) left arm and left leg (LaLl); (5) right arm and left leg (RaLl); (6) left arm and right leg (LaRl) (see also Table 2).

While leg–leg PCI was presented and used before [31,33,40,41], we herein present a generalization of the use of PCI to any other limb pairing.

Similar to the leg–leg PCI, the PCI calculation of the other five pairs of limbs was performed as follows:

For arm–arm PCI, the series of φ was calculated by identifying one’s arm MFS time point relative to the cycle of the second arm (i.e., the time between consecutive MFSs of the second arm). In this case, the ideal value of φ is 180° (anti-phase). Please note that the choice of the side that defines a cycle (i.e., MFS to MFS) is arbitrary.

For ipsilateral arm–leg PCI, the gait events used are the HS and the MFS on the same side. The MFS time point defines the φ value with respect to the gait cycle duration between two consecutive heel strikes (arm relative to leg; AreL). Also, here we consider the ideal value of φ to be 180°. In this case, a series of φ values can also be calculated by referring to the timing of the HS to the cycle duration defined by MFS to the consecutive MFS (leg relative to arm; LreA).

For contralateral arm–leg PCI, the gait events used are the HS of the leg and the MFS of the contra-lateral arm. In this case, the ideal φ is 0°. In order to conform with the PCI formulation (recall Equation (1)), we generated a series of ‘corrected’ φ’= φ + 180° (from here on, we will refer to both φ’ and φ as φ). In this case, a series of φ values can be calculated by referring the timing of the HS to the cycle duration defined by MFS (LreA; stands for leg relative to arm) or vice versa (AreL; stands for arm relative to leg). See Supplementary Materials, Section C, for ad hoc confirmation on the phase value of 180^0^ as the ‘ideal’ φ value for all possible limbs’ pairing.

As previously described in detail for the legs, in all limb-pairing cases the series of φ values generated using one limb cycle is reciprocal (with reference to 180°) to the series of φ values calculated in reference to the second limb’s cycles. Thus, in this study, we calculated PCI as the mean value of both PCIs, each calculated based on one of the two φ values vectors. For example, in the case of arm–arm PCI, we calculated the RreL PCI using the φ vector computed from the right arm MFS relative to the cycles defined by the left arm MFS, and the LreR PCI using the φ vector computed from the left arm MFS relative to the cycles defined by the right arm MFS. These two values of PCI were averaged to yield the arm–arm PCI (recall Table 2).

### 2.5. Four-Limb PCI: Q-PCI

Herein, we present how to calculate the new measure, Q-PCI, proposed in this study.

#### 2.5.1. Generating Unified φ Vector for Calculating Q-PCI

After calculating the six values of PCI that quantify the coordination between all possible limb pairings between the four limbs, we united all six φ vectors in order to define an index that measures the total gait coordination of the limbs, herein termed quadruple-PCI (Q-PCI). As explained before, there are two alternatives to generate the φ vector from each inter-limb pairing. We detail below the scheme of choosing the values that will ‘enter’ the united φ vector from each inter-limb pairing. As in the case of single inter-limb PCI, there are two alternative schemes to generate the united φ vector based on which Q-PCI can be calculated. One scheme is based on referring the φ values to the cycle of the right leg (right HS anchor) and one is based on referring the φ values to the cycle of the left leg (left HS anchor). It is important to note that anchoring to the right HS is equivalent to anchoring to the left arm MFS, and likewise, anchoring the left HS is equivalent to anchoring to the right arm MFS. This is because the HS of one leg occurs simultaneously with the MFS of the contralateral arm. However, for each of the schemes, there is one instance where neither the anchoring HS nor the contralateral MFS are involved. When anchoring to the right HS, the RaLl pairing does not include the right HS or the left MFS, here we chose the anchor of the right MFS as an anchoring event for the gait cycle (see last line in Table 3). The corresponding rule was applied for the scheme in which the left MFS served as an anchoring event (see line 5 in Table 3). Table 3 details which φ values enter the united φ vector in each of the ‘unification’ schemes.

#### 2.5.2. Calculating Q-PCI

For each sachem, we used the unified φ vector to calculate Q-PCI using Equation (1). Then, we averaged both Q-PCI values and received the united index that represents four-limb coordination.

#### 2.5.3. φ Values for Six Pairs of Limbs

**Figure 4:**
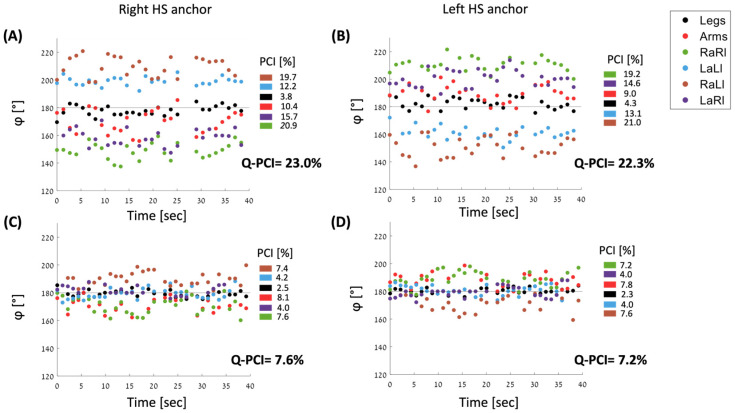
φ values calculated for all six limb pairs for each gait cycle for 40 s. Top panels (**A**,**B**) show a participant with high values of PCI (i.e., less coordinated, values are indicated near the color key). Bottom panels (**C**,**D**) show a participant with low values of PCI. Panels A and C show the phi values calculated relative to the right step and the left panels (**B**,**D**) show the φ values calculated with the left leg stepping as an anchor. The two types of φ vectors (i.e., left and right anchored) ‘mirror’ each other with reference to the φ = 180° line (horizontal black line). For example, in (**A**), the φ values of the RaLl relative to the right step are around 200° and relative to the left step (**B**) around 160°.

Figure 4 presents the φ values of all six pairs of limbs calculated for each gait cycle, for two different participants presenting different four-limb coordination values.

### 2.6. Statistical Analysis

Statistical analysis was performed in MATLAB (version R2021b, MathWorks, Natick, MA, USA) and in Jasp software (version 0.18.3).

#### 2.6.1. Descriptive Statistics

To verify the suitability of parametric statistics, Shapiro–Wilk normality tests were performed for the PCI variable for each limb pairing for each group (YA and OA). This procedure showed that PCI data were not normally distributed (*p* < 0.03). The data, then, were log-transformed and normality was confirmed (*p* > 0.18). Thus, analysis of variance (ANOVA) using the log-transformed data was performed for comparative analysis. Illustrations and tables depict the original, un-transformed, data.

Summary measures are reported as the median and quartile range for the PCI of the six limb pairings and for the Q-PCI.

#### 2.6.2. Statistical Comparisons

To evaluate the effects of limb pairing and group, we performed repeated measures ANOVA with 7X2 levels: six limb pairings, one Q-PCI (within participants), and two age groups (YA and OA; between participants). If the within-participant or between-participant results were significant, post hoc analyses were performed correcting for multiple comparisons (Holm correction). The Eta square coefficient was used to describe the effect size.

Correlation analyses (Pearson and Spearman) were performed to assess the relations between the coordination of different limb parings. We also employed stepwise regression analysis to identify the limb pairings that best accounts for the Q-PCI. Additionally, we studied the association between PCI values of limb pairings that involved arms and the arm swing amplitude using Spearman correlation.

## 3. Results

### 3.1. Comparisons between PCI Values

**Figure 5:**
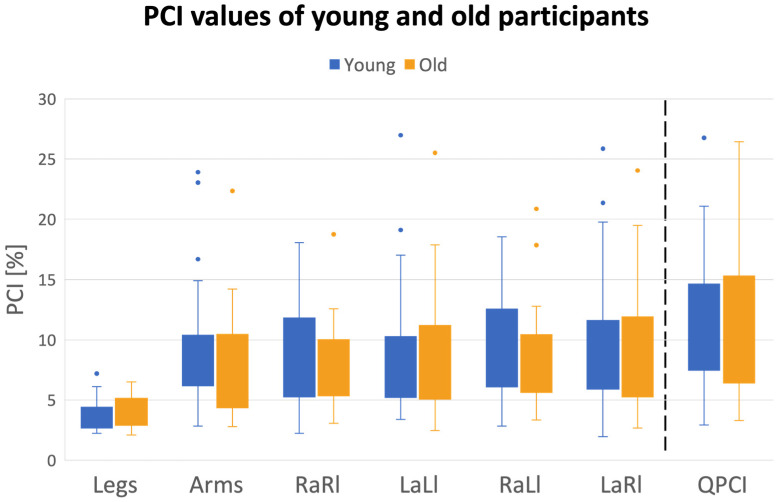
Box plots of different types of PCI are plotted for young and older adults (see key). A horizontal line within the box indicates median value, and upper and lower borders of the box indicate first and third quartiles. ‘Whiskers’ present the range when excluding the outliers. Note: outliers were not excluded from the statistical analysis since the data were log transformed. The dashed vertical line differentiates between the PCI of all limbs pairings and the Q-PCI.

Table 4 and Figure 5 detail the different values of the different types of PCI in both groups. Repeated measures ANOVA revealed a significant limb pairing effect (F = 85.05, *p* < 0.001, ɳ^2^ = 0.25). Group effect was not statistically significant (F = 0.77, *p* = 0.38, ɳ^2^ = 0.006). Post hoc analysis showed that the PCI values of the legs and Q-PCI were significantly different from all the other PCI values (*p* < 0.005 for both legs and Q-PCI), with the former presenting the smallest, and the latter the largest, values of PCI. PCI values for all other limb pairings did not differ from each other (*p* > 0.34). See Supplementary Materials, section D for the non-parametric test results.

Since we did not find a difference between the PCI values of the YA and OA, correlation analyses were conducted considering the data from both groups combined (i.e., 71 individuals).

### 3.2. Correlation between the Q-PCI and the PCI of all Limbs’ Pairings

**Figure 6:**
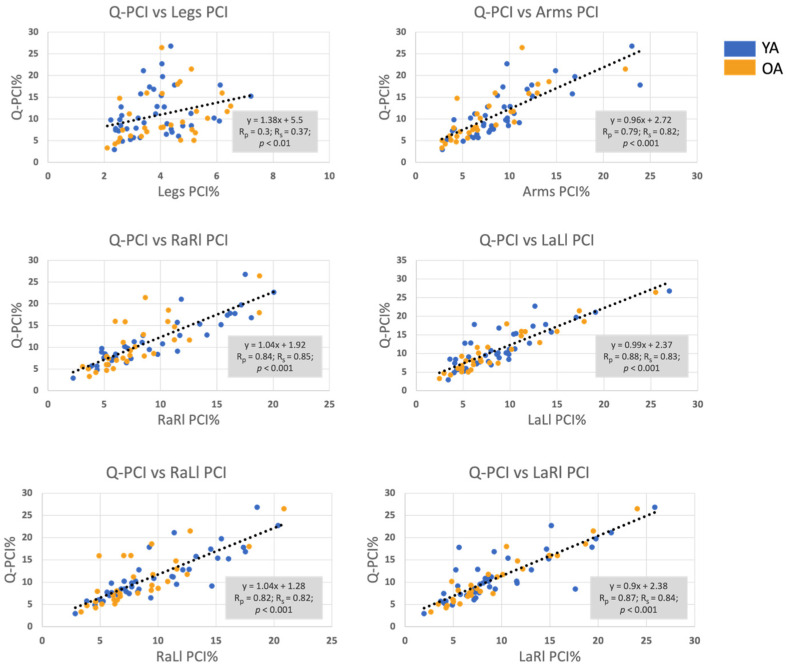
The correlations between the Q-PCI and the PCIs of each limb pairing. Blue dots represent the PCI of the YA and orange dots represent the PCI of the OA. The linear fit (black dashed line) was computed from PCI values of the YA and OA combined. R_P_: Pearson’s R; R_S_: Spearman’s R.

The correlation coefficients between PCI values obtained for limb pairings that involve arms and the Q-PCI indicate strong correlations (Pearson’s R > 0.79; Spearman’s R > 0.82, *p* < 0.001), while the correlation between the leg–leg PCI and the Q-PCI was found to be moderate (Pearson’s R = 0.3; Spearman’s R = 0.37, *p* < 0.01; see Figure 6).

### 3.3. Correlation Matrix

In order to evaluate the relations between the PCI of all limbs’ pairings, we calculated a PCI correlation matrix that shows the correlation between all PCI types, see Table 5.

### 3.4. Stepwise Regression

A multiple stepwise regression analysis was used to test which of the limb pairing PCIs predicts the Q-PCI significantly. This resulted in a significant model (F(30,70) = 392.17, *p* < 0.001, R^2^ = 0.94) consisting of three predictors: LaLl (β = 0.41, *p* < 0.001), RaRl (β = 0.48, *p* < 0.001) and LaRl (β = 0.2, *p* = 0.004).

### 3.5. How Arm-Related PCI Affects Leg–Leg PCI

In a post hoc analysis, we modified the Q-PCI and excluded φ values arising from the right leg–left leg coordination (recall the methods for Q-PCI). We then conducted correlation analysis between this modified Q-PCI and the leg–leg PCI. A significant correlation was found (R_S_ = 0.44; *p* < 0.002).

### 3.6. Effect of Arm Swing Amplitude on PCI Values

**Figure 7:**
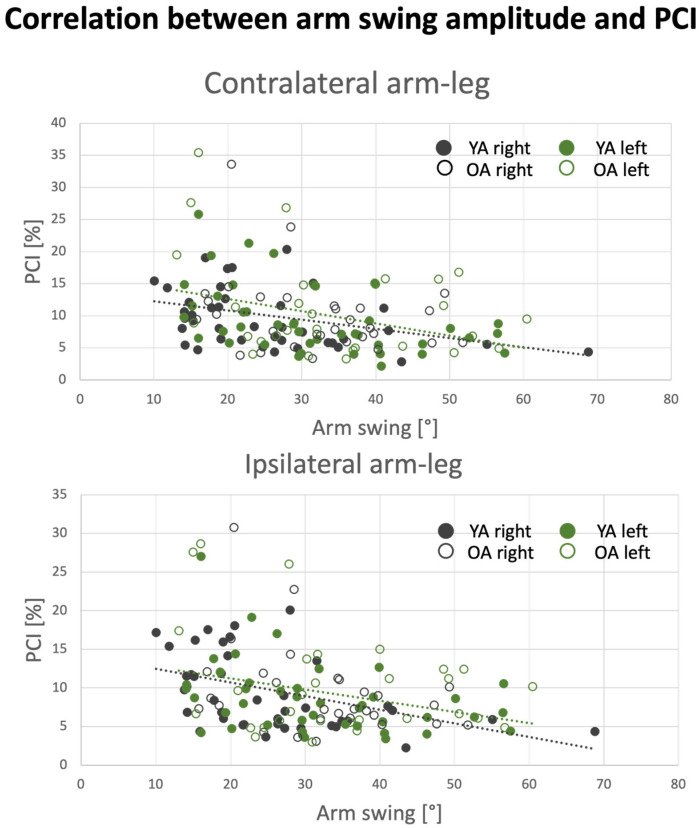
Correlations between PCI and arm swing amplitude. PCI values are based on the corresponding arm. Top panel shows the ipsilateral arm–leg pairings (black dots: right arm–right leg, linear fit: y = −0.2x + 13.5; green dots: left arm–left leg, linear fit: y = −0.1x + 11.9). Bottom panel shows the contralateral arm–leg pairings (black dots: right arm–left leg, linear fit: y = −0.1x + 12.8; green dots: left arm–right leg, linear fit: y = −0.14x + 13.7). Filled dots represent the young population and blank dots represent the old population. The linear fit was computed from the young and old adults combined.

We assessed the influence of arm swing amplitude on the coordination of different limb pairings for the right arm (mean arm swing amplitude ± SD: YA: 25.6 ± 12^0^; OA: 31.8 ± 10^0^) and for the left arm (mean arm swing amplitude ± SD: YA: 31.6 ± 12.5^0^; OA: 34.1 ± 13^0^). We found that there was a negative correlation between the arm swing amplitude and the corresponding PCI values, meaning that a higher arm-swing amplitude is associated with more coordinated walking (see Table 6 and Figure 7). Leg–leg PCI values were not correlated with arm swing amplitude (*p* ≥ 0.07).

## 4. Discussion

Under the overreaching objective of investigating the temporal coordination between the rhythmic movements generated by all four limbs during walking, we aimed here: (1) To use and adapt the PCI metric [31] from leg–leg coordination (i.e., left–right stepping phase coordination) to all possible pairings between any two limbs (arms and legs); (2) To evaluate a new metric, Q-PCI, that evaluates four-limb coordination as a whole and (3) to study the effect of aging on four-limb coordination. Our observations are based on the functional level, i.e., movements documented by body fixed sensors, as opposed, for example, to locomotion-related muscular activity (e.g., [28,42]). Using bodily attached sensors provides information on the spatiotemporal aspects of cyclic movements, including events (e.g., HS) that allow further computation of coordination.

### 4.1. Summary of Findings

PCI analysis revealed that the most coordinated inter-limb rhythmic movements are the leg–leg pairing, i.e., the left–right stepping movements (c.f., Figure 5, and Table 4). All limb pairings involving arm–leg coordination (four pairings) and arm–arm coordination presented similar levels of coordination as reflected by the corresponding PCI values. As might be expected, the Q-PCI metric had the higher value since it involves phasing values obtained from every possible inter-limb pairing. Importantly, Q-PCI was found to be significantly correlated with all other PCI values, indicating levels of coordination between other limb pairings. However, stepwise regression analysis showed that the pairs of limbs whose coordination explained most of the variability in the Q-PCI were: RaRl, LaLl and LaRl. Our findings also point to the fact that swinging movements of the arms during walking are better coordinated with leg stepping (either the contra- or ipsi-lateral leg) when the swinging amplitude is increased (c.f., Figure 7). Finally, in our samples, YA and OA did not differ in terms of the levels of inter-limb coordination, as reflected by the different PCI measures (c.f., Figure 5).

### 4.2. Arm Swinging Related PCI Values

The most striking result of this study is that leg–leg PCI is about half of the PCI values that involve the arms. This finding might suggest that the neuronal substrate that coordinates the timing of the leg–leg stepping phasing is the most ‘precise’; it consistently and accurately generates a phasing of 180^0^ between cyclic stepping movements, as compared to neural substrates that drive the coordination between arm swinging and either leg stepping (left or right) and/or arm swinging of the contra lateral arm. However, in a previous study where arm–arm swinging PCI was calculated, values were similar to or lower than the leg–leg stepping PCI [43], but never higher. Nevertheless, that study only investigated the coordination between the arms and the legs separately, and not arm–leg coordination. To the best of our knowledge, the PCI metric has hardly ever been used to quantify arm–arm coordination (but see, [44]). Han and Paul calculated a smartwatch gait coordination index based on the PCI, and found similar arm–arm and leg–leg coordination values [45].

What might be the reason for these differences in phasing coordination levels (i.e., as reflected by different PCI values, see Table 4) between different limb pairings? We speculate that three factors may be involved. From the functional point of view, since gait serves to progress the walker in space, alternation of stepping movements is the most effective pattern for this progression (e.g., as opposed to ‘jumping’ or ‘galloping’). Arm swinging, on the other hand, does not directly affect spatial progression. In fact, effective locomotion can be achieved even when the arms are restricted, having minimal or no swing [4,46,47]. Therefore, during the development of the human locomotor neural system, anti-phased leg stepping was re-enforced, but phasing of arm swing movements relative to the other limbs was reinforced to a lesser extent, which is reflected in higher variability and reduced accuracy (i.e., a larger PCI value).

As for the second explanation for larger PCI values when arm swinging is involved, physiologically, both in animal models (e.g., [48]) and in human studies - for a review [21]) that focus on locomotion related to four-limb coordination, the contribution of sensory feedback to inter-limb coordination is emphasized. Cutaneous receptors, muscle spindles, and joint receptors (e.g., Golgi tendon organs) provide cyclic input that drives both spinal and supra-spinal circuits that mediate locomotion. It is reasonable to assume that the overall sensory influx from the lower limbs is greater than that arising from the upper limbs. This is true because muscle mass, weight bearing, and tactile inputs (e.g., from the floor during stance) are greater in the lower limbs as compared to the upper limbs. Possibly, a larger sensory influx contributes to more coordinated movements since the signals in the relevant locomotion circuits are amplified due to the relatively larger sensory ‘boost’ arising from the legs as compared to the arms.

Finally, a third possible factor behind lower inter-limb coordination when the arms are involved are the differences in mechanical constraints imposed on the event characteristics between leg and arms (HS and MFS, respectively) during HS. Because the feet are in contact with the floor, there is not much room for physical variability, while the arms are always free to ‘wiggle’ about when swung forward, increasing the potential room for variability and consequently decreasing the consistency of phasing.

### 4.3. Q-PCI

The methodology that we used to develop the Q-PCI metric is consistent with that used for developing the original leg–leg PCI [31]. Aspects such as the anchoring method (c.f., Figure 4) and adjusting the vectors of inter-limb φ values around an ideal figure of 180^0^ were preserved. Indeed, it was validated here that Q-PCI is correlated with all other inter-limb coordination PCIs (Figure 6 and Table 5), reinforcing our notion that Q-PCI provides a reliable evaluation of inter-limb coordination abilities during walking. On the other hand, our stepwise regression analysis only partially supports this assertion since only three of the inter-limb PCI values explain most of the variability of Q-PCI: RaRl, LaLl and LaRl, all involving, however, arm–leg coordination. One peculiarity is also the fact that leg–leg PCI is not among these three measures. This observation may be related to the lower within and between subject variability, reflected in the lower leg–leg PCI, reiterating the relevance of an additional metric, i.e., the Q-PCI. Thus, we propose that in addition to the Q-PCI, for a comprehensive picture of four-limb coordination, the entire inter-limb pairings PCI values matrix is needed (as in Figure 4 and Figure 5, Table 4).

The Q-PCI was developed based on a spinal cord model presented by Ijspeert et al. (2007) [49]. The model, which was implemented in a salamander robot, integrates CPGs for both limb and axial movements and illustrates how limb and body oscillatory centers interact to coordinate different modes of locomotion, such as swimming and walking. The core approach of our work is to quantify the coordination between the various “oscillators”; in our case, the Q-PCI is based on the CPG structures. When looking at the combined coordination function of all “oscillators” (Q-PCI) and at individual inter-limb coordination function of the “oscillators” (e.g., leg–leg), we enhance our understanding of inter-limb coordination during human walking. 

### 4.4. Relationship between Arm Swinging Coordination and Leg Stepping Coordination

There is no debate on the fact that upper and lower limb movements are linked and affected by each other; this is shown in multiple previous studies (e.g., [21,28]). This is also supported by our results, as they show that all PCI values are correlated with each other (e.g., legs PCI with arms PCI, ipsilateral arm–leg PCI with contralateral arm–leg PCI etc.). Further, when we generated a combined PCI metric that included all phasing relations but for that of the leg–leg coordination in our post-hoc analysis, we found that leg–leg PCI is associated with this ad hoc metric that includes arm swinging. These results could also be maintained in the context of the directional influence of lower-upper limb coordination studied by Zehr et al. [21] and Weersink et al [28,29].

Finally, PCIs involving arm swinging were correlated with arm swing amplitude (Table 6 and Figure 7), but leg–leg PCIs did not reach a statistically significant association with the arm swing amplitude (i.e., *p* ≥ 0.07). Despite this latter finding, the data presented in this study support the notion that arm-swinging inter-limb coordination is linked with lower limb stepping coordination.

### 4.5. Effect of Aging

In the present study, age was not a significant effect when all PCI metrics were considered (Table 4 and Figure 5). This is in contrast to some of the earlier studies, including the original PCI paper [31], that did indicate coordination deterioration with ageing (see more, e.g., [43]). Additionally, in a recent analysis, we proposed that a significant change in PCI values occurs around the age of 70 [32], and one study even suggested that only among older-old (i.e., above 85 years old) is leg–leg PCI different [50]. These inconsistent results require a deeper investigation with more normative data (i.e., data of old adults over 70 and 85 years old) to address the role of aging in the different inter-limb coordination capabilities.

### 4.6. Limitations and Future Directions

For calculating Q-PCI and any inter-limb pairing related to PCI, we relied on an ‘axiom’ that, during gait, normal coordinated rhythmic movements of the limbs are either 180^0^ phased apart from each other (i.e., leg–leg, arm–arm, ipsi-lateral arm–leg), or that their defining events (i.e., HS, and MFS) occur simultaneously (0^0^, MFS of one arm with the contralateral HS). In the latter case, the collected φ values are shifted by adding a constant of 180^0^ (recall *Methods*). The distributions of individual participant mean φ values (c.f. Section C in the Supplementary Materials) were around these phase values. However, it might be the case that for some individuals, the inter-limb phasing default is shifted from 180^0^/0^0^ (c.f. Figure S4A, in the Supplementary Materials). In this case, the value of PCI is relatively increased due to the variable φ_ABS_ (which includes absolute differences from the ideal figure of 180^0^), but it might be that this slight phasing shift is not inferior to the 180^0^/0^0^ default. This limitation has a minimal effect when observing cohorts, and more research and normative data collection in healthy and diseased populations is needed in order to address this subtle effect.

The present study is based on TM data. TM vs. over-ground walking is a more reactive type of walking, reducing variability and decreasing potential age-differences in muscle activation patterns and kinematics [51,52,53]. Indeed, we used self-paced TM, and these effects are more evident during fixed-speed TM, but even the self-paced TM walking may result in a lower variability type of walking. Future studies may utilize inertial sensors data to examine the four-limb coordination during over-ground walking and increase the external validity of this study.

The constitution of segmental masses influences the moment of inertia of body segments, affecting the segments acceleration and deceleration. Therefore, a dynamic analysis of center of mass, kinetic/potential energy and gesticulation energy might be informative and could potentially enrich the analysis and evaluation of four-limb coordination.

From clinical perspective, arm swing amplitude was found to be correlated with the arm–leg coordination (ipsi-lateral and contra-lateral), when the higher the arm swing amplitude, the higher the coordination. Sensory biofeedback to the arm swing amplitude can be beneficial to modulate the arm swing and hence reach a higher arm–leg coordination.

## 5. Conclusions

This research presented a comprehensive assessment of gait coordination and a novel method for quantifying four-limb coordination: the Q-PCI. This measure is based on quantifying the inter-limb phasing during locomotion. The Q-PCI was found to be significantly correlated with the PCI values of all possible limb pairings with a higher correlation to the PCI values of the pairs of limbs that involve arms. This suggests that arm swing coordination is linked with leg stepping coordination and highlights the importance of evaluating quadruple gait coordination.

## Figures and Tables

**Table 1:** Demographics.

	N	Age [Years], Mean (Range) *	F/M
YA	41	27 (21–36)	20/21
OA	30	70 (61–77)	10/30

* OA significant difference from YA (*t*-test; *p* ≤ 0.05). Ratios were compared using Chi-square tests. YA—young adults; OA—older adults.

**Table 2:** Limbs coupling.

Pair of Limbs	Leg–Leg	Arm–Arm	RaRl	LaLl	RaLl	LaRl
Phase Type	Anti-phase	Anti-phase	Anti-phase	Anti-phase	In-phase	In-phase
Coordination type	Contra- lateral	Contra- lateral	Ipsi- lateral	Ipsi- lateral	Contra- lateral	Contra- lateral

RaRl-right arm and right leg; LaLl-left arm and left leg; RaLl-right arm and left leg; LaRl-left arm and left leg.

**Table 3:** φ vectors taken to calculate the Q-PCI.

Pair	For Q-PCI Based on Right HS Anchor	For Q-PCI Based on Left HS Anchor
Leg–leg	LreR	RreL
Arm–arm	RreL	LreR
Right arm–right leg	AreL	LreA
Left arm–left leg	LreA	AreL
Left arm–right leg	AreL	LreA
Right arm–left leg	LreA	AreL

LreR-left relative to right cycle; RreL-right relative to left cycle; AreL-arm relative to leg cycle; LreA-leg relative to arm cycle.

**Table 4:** PCI values for YA and OA: median (1st qrt., 3rd qrt.).

	Legs PCI%	Arms PCI%	RaRl PCI%	LaLl PCI%	RaLl PCI%	LaRl PCI%	Q-PCI%
**YA**	3.76 (2.8, 4.4)	8.22 (6.3, 10)	7.38 (5.3, 11.8)	7.95 (5.3, 10.4)	8.34 (6.4, 12.7)	8.08 (6.4, 11.6)	9.75 (7.6, 15.2)
**OA**	4.08 (3.0, 5.1)	6.5 (4.4, 10.3)	6.93 (5.3, 9.2)	6.73 (4.9, 10.9)	7.34 (6.1, 9.9)	7.15 (5.1, 11.4)	8.35 (6.2, 14.3)

YA-young adults; OA-older adults; Qrt-quartile.

**Table 5:** Correlation matrix.

	Leg	Arms	RaRl	LaLl	RaLl	LaRl	Q-PCI
Legs	-						
Arms	0.47	-					
RaRl	0.43	0.73	-				
LaLl	0.28	0.63	0.57	-			
RaLl	0.41	0.71	0.91	0.58	-		
LaRl	0.28	0.69	0.60	0.87	0.57	-	
Q-PCI	0.37	0.82	0.85	0.83	0.82	0.84	-

Spearman correlation. All correlations are significant with *p* < 0.02.

**Table 6:** Correlation between arm swing amplitude and the corresponding PCI.

Arm Swing Amplitude	Pair of Limbs	Correlation (Spearman’s’ Rho)
Right hand	Arm–arm	−0.50 **
	RaRl	−0.43 **
	RaLl	−0.36 *
Left hand	Arm–arm	−0.38 *
	LaLl	−0.25 *
	LaRl	−0.37 *

N = 71 as data from young and old were combined. See Supplementary Materials Section E for the correlation of each group separately. * *p* < 0.05; ** *p* < 0.0001.

## Data Availability

The raw data supporting the conclusions of this article will be made available by the authors on request.

## Supplementary Materials

The following are available online at https://www.mdpi.com/article/10.3390/s24186105/s1, For the Methods section, subsection Participants: Table S1: Participants by partition to the study protocols used for collecting data for the current study. For the Methods section, subsection PCI definition and calculation: Table S2: φ values of legs analysis; Figures S1–S4: Frequency distribution of individual mean φ values. For the Results section, subsection Comparison between PCI values: a description of the results of the non-parametric analysis. For the Results section, subsection Effect of arm swing amplitude on PCI values: Table S3: Correlation between the arm swing amplitude and the correspondence PCI [54,55,56,57,58,59,60].
