# Supplementary material for: A New Measure for Quantifying Four-Limb Coordination of Human Gait Based on Mobility Sensors
Source: Sensors (Basel). 2024 Sep 21;24(18):6105. doi: 10.3390/s24186105 (PMC11435969; doi:10.3390/s24186105)
Supplement: Supplementary file 1 [file sensors-24-06105-s001.zip › sensors-3022234-supplementary.pdf]

Supplementary material for:
A new measure for quantifying four-limb coordination of human gait based on mobility sensors

A. Supporting material for the *Methods* section- Subsection: *Participants*

Table S1: Participants by partition to the specific experimental protocol used for collecting data for the current project

<table>
<tr><th>Title of research</th><th>YA</th><th>OA</th></tr>
<tr><td>The effect of incongruent walking on gait speed, muscle activation and gravity perception [1–3]</td><td>41</td><td>17</td></tr>
<tr><td colspan="3">Walking procedure: Participants walked on a self-paced treadmill, in their own comfortable pace, until reaching steady state velocity. Then, different physical inclinations were introduced (i.e., the actual surface pitch; downhill, uphill, or level walking) and in different virtual inclinations (i.e., the visual surface pitch that was projected on a large scale virtual reality system). The conditions were randomized and for the present study, we included walking trials that combined physical level walking and virtual level walking. This experiment was done using a dome-shaped system (CAREN High End, Motek Medical, The Netherlands).</td></tr>
<tr><td>Algorithms development for the analysis of physiological networks for fall prevention in elderly subjects with and without neurological disease using virtual reality environments [4,5]</td><td>-</td><td>6</td></tr>
<tr><td colspan="3">Walking procedure: As part of a larger walking protocol participants walked on a self-paced treadmill (split-belt treadmill), at their own comfortable pace until reaching steady state velocity, then physical perturbations were introduced (by moving the platform in which the treadmill is embedded, or by sudden stop/acceleration of one of the belts of the treadmill. After the introduction of a perturbation, the person regained a steady state walking before next presentation of perturbation. We included only the gait segments where a steady state was reached and maintained for at least 30 seconds prior to introducing the perturbations. This experiment was done using a dome-shaped system (CAREN High End, Motek Medical, The Netherlands).</td></tr>
<tr><td>Differential gait adaptation patterns in Parkinson's disease - a split-belt treadmill study [6]</td><td>-</td><td>4</td></tr>
<tr><td colspan="3">walking procedure: The system used in this protocol includes split-belt treadmill that can be operated in self-paced mode. The main part of the protocol included the introduction of split-belt conditions. To individualize the procedure, we first determined the preferred walking speed, but letting the participant reaching and maintaining a steady state walking speed in his/her own preferred pace. These gait segments of steady-state self-paced walking speed were included in the present study. This experiment was done using a semi-immersive virtual system (V-GAIT; Motek Medical, The Netherlands).</td></tr>
<tr><td>Studying the dynamics of neurophysiological networks' interactions predisposing freezing of gait in Parkinson's disease with virtual reality [7]</td><td>-</td><td>3</td></tr>
<tr><td colspan="3">walking procedure: As part of a larger walking protocol, participants walked on a self-paced treadmill while overcoming virtual obstacles (e.g., narrow door passage, turns) that were projected on a large-scale virtual reality system. In the present study, we included walking trials that did not include obstacles (i.e., straight walking in a virtual corridor) . This experiment was done using a dome-shaped system (CAREN High End, Motek Medical, The Netherlands).</td></tr>
<tr><td rowspan="2">Total:</td><td>YA</td><td>OA</td></tr>
<tr><td>41</td><td>30</td></tr>
<tr><td colspan="3">YA- healthy young adults; OA – healthy older adults</td></tr>
</table>

Supplementary material for:
A new measure for quantifying four-limb coordination of human gait based on mobility sensors

B. Supporting material for the *Methods* section- Subsection: *PCI definition and calculation – reciprocal relation between LreR and RreL schemes for extracting φ values*

We herein demonstrate that the φ values obtained from LreR analysis (left leg relative to right leg) 'reciprocate' to the φ values obtained from RreL (right leg relative to left leg) analysis with respect to 180°. Table S2 depicts the mean φ values of 10 random participants in the LreR analysis compared to the RreL analysis of the legs.

**Table S2: φ values of legs analysis**		
**Participants**	**LreR φ (mean ± SD)**	**RreL φ (mean ± SD)**
Sub. 1	182.6	177.9
Sub. 2	179.9	180.1
Sub. 3	184.9	174.1
Sub. 4	178.2	181.8
Sub. 5	176.6	183.3
Sub. 6	184.2	175.3
Sub. 7	180.8	179.4
Sub. 8	182.0	178.0
Sub. 9	178.5	181.8
Sub. 10	183.2	176.5

C. Supporting material for the *Methods* section- *PCI definition and calculation- The ideal phasing relation between all possible limbs pairings*

The calculation of PCI is based on computing two variables φ_{CV} and φ_{ABS} (see Eq. 2 in the body of the article). The latter expression provides information on the accuracy of generating the phasing relation between the cyclic movements of two limbs. This accuracy is measured by calculating, for each gait cycle, the absolute difference between the generated inter-limb phasing and the presumed 'phasing relation' (i.e., the 'ideal' phasing). In all six possible limbs' pairing, this ideal phasing relation is either 180º or 0° (see Figure 3 in the main text). Herein, we empirically validate these values. Data are taken from 41 young adults (see *Participants* in the main article), presumably reflecting normative data.

Legs stepping phasing relation - 180º

We measure the heel strike (HS) timing of one heel with respect to the HS cycle of the other leg. Here, we arbitrarily choose to refer the stepping phases of the left leg with respect to the right leg (φ) and calculated for each participant the mean value of φ for all gait cycles. Across participants (n=41), the mean value (± SD) of φ is 180.9 ± 2.2º (see Figure. S-1).

Supplementary material for:
A new measure for quantifying four-limb coordination of human gait based on mobility sensors

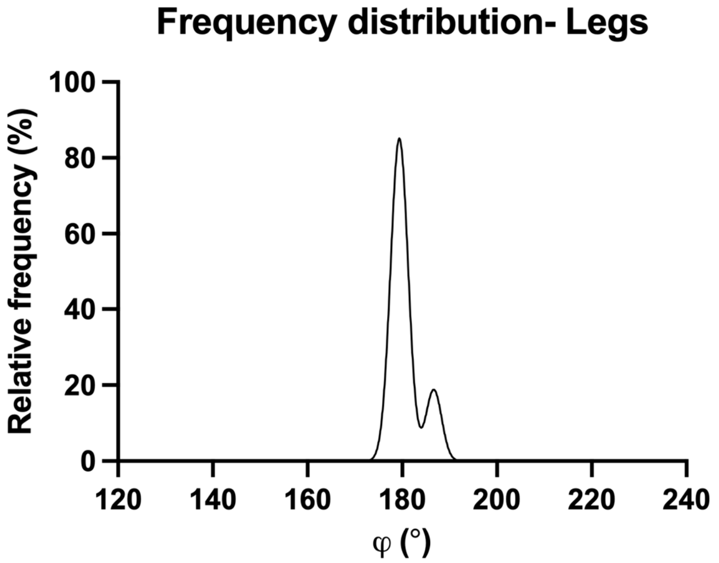

Figure S-1: Frequency histogram of individual mean φ values for leg-leg stepping phasing relations. Statistical analysis reveals that the distribution is normal (Shapiro-Wilk test; p=0.052). Skewness is 0.61 and Kurtosis is 2.4.

Arms swinging phasing relation - 180°.

We measure the maximal forward swing (MFS) timing of one arm with respect to the MFS timing of the other arm (cycle duration defined as the duration between MFS events). Here we arbitrarily choose to refer the swinging phases of the left arm with respect to the right arm (φ) and calculated the mean value of φ for all gait cycles for each participant. Across participants, the mean value (± SD) of φ is 179.8 ± 9° (see Fig. S-2).

Supplementary material for:
A new measure for quantifying four-limb coordination of human gait based on mobility sensors

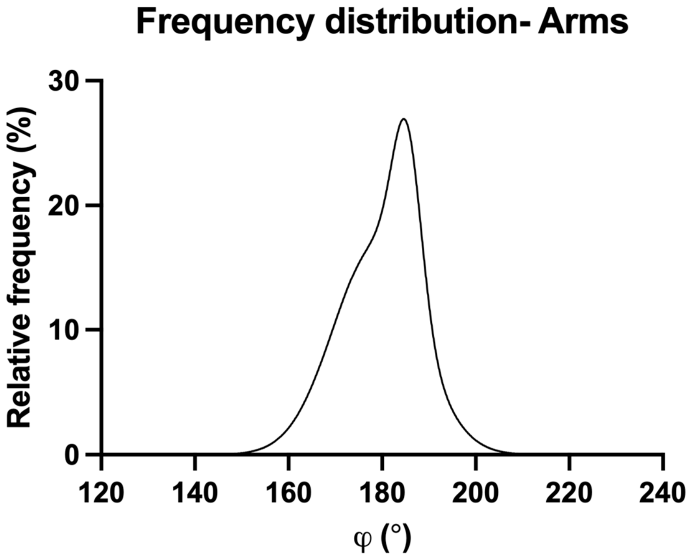

Figure S-2: Frequency histogram of individual mean φ values for arm-arm swinging phasing relations. Statistical analysis reveals that the distribution is normal (Shapiro-Wilk test; $p=0.55$). Skewness is -0.51 and Kurtosis is 0.13.

Arm swinging – leg stepping phasing relation

Here, we have two cases of ipsilateral arm swinging – leg stepping phasing, one for each side, and two cases of contra-lateral arm swinging – leg stepping coordination, right arm swinging with left leg stepping, and left arm swinging with right leg stepping.

For the first type, ipsi-lateral arm swinging- leg stepping phasing, we assign the value of 180º as the ideal phasing relation.

We measure the MFS timing of one arm with respect to cycle defined by the duration between two consecutive heel strikes generated by the leg on the same side of the body. Across participants, the mean value (± SD) of φ is 176 ± 10.7º for the right side of the body (see Figure S-3A), and 176.7 ± 11.7º for the left side of the body (see Figure S-3B).

Supplementary material for:
A new measure for quantifying four-limb coordination of human gait based on mobility sensors

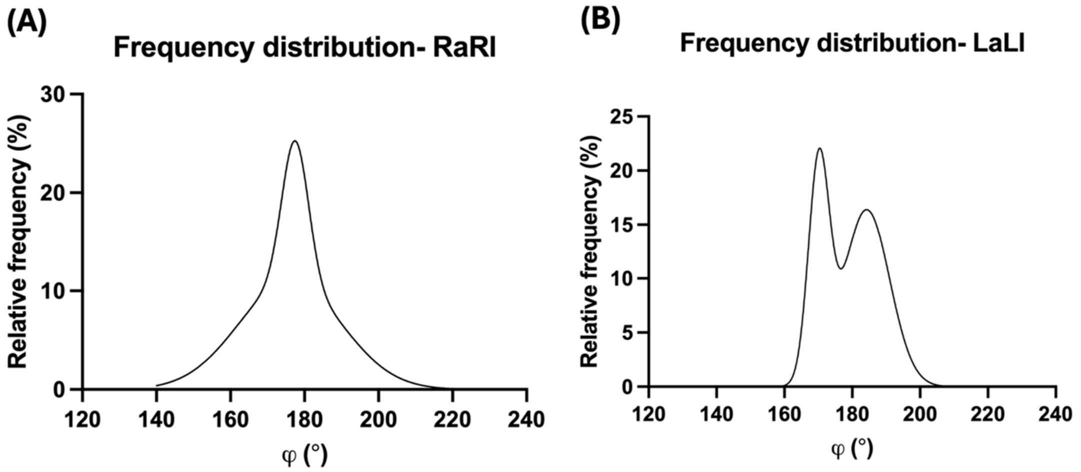

Figure S-3: A. Frequency histogram of individual mean φ values for right arm swinging – right leg stepping phasing relations. Statistical analysis reveals that the distribution is normal (Shapiro-Wilk test; $p=0.6$). Skewness is -0.33 and Kurtosis is 0.27.

B. Same for the left arm swinging – left leg stepping phasing relations. Statistical analysis reveals that the distribution is normal (Shapiro-Wilk test; $p=0.36$). Skewness is -0.5 and Kurtosis is 0.25.

For the second type, of arm swinging – leg stepping coordination, i.e., contralateral arm swinging- leg stepping phasing, we assign the value of 0° as the ideal phasing relation. That is, since the timing of the MFS events of the arm occurs roughly simultaneously with the HS of the contralateral leg. We measure the MFS timing of one arm with respect to cycle defined by the duration between two consecutive heel strikes generated by the leg on the other side of the body. In order to conform with PCI formulation (recall Eq. 2, in the body of the article), we generate a series of 'corrected' $\varphi' = \varphi + 180°$ (from here on, we will refer both to φ' and φ as φ). With the corrected **φ,** across participants, the mean value (± SD) of φ is 176.8 ± 11.1° for the right arm – left leg coordination (see Fig. S-4A), and 175.8 ± 12.1° for the left arm – right leg coordination (see Figure S-4B).

Supplementary material for:
A new measure for quantifying four-limb coordination of human gait based on mobility sensors

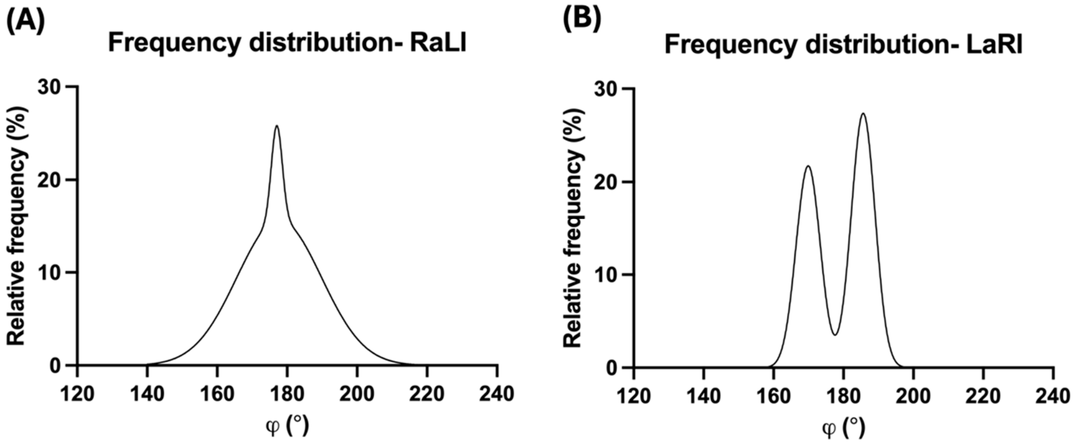

Figure S-4: **A.** Frequency histogram of individual mean φ values for right arm swinging – left leg stepping phasing relations. Statistical analysis reveals that the distribution is normal (Shapiro-Wilk test; p=0.9). Skewness is -0.27 and Kurtosis is 0.1.

B Same for the left arm swinging – right leg stepping phasing relations. Statistical analysis reveals that the distribution is normal (Shapiro-Wilk test; p=0.3). Skewness is -0.37 and Kurtosis is 0.38.

D. Supporting material for the *Results* section- *Subsection: Comparison between PCI values*

Non-parametric analysis:

Since the data were not normally distributed, Friedman test was also performed over the original data to assess the differences in PCI values among different conditions for both young adults and old adults. The results of the non-parametric analysis resemble the results we obtained in the parametric analysis (over the log-transformed data):

The analysis revealed a significant difference in PCI values for both groups (young adults: $\chi2=113.57$, $p<0.001$; older adults: $\chi2=80.21$, $p<0.001$). Post hoc analysis indicated that the leg-leg PCI was significantly different from all the other PCI values ($p<0.005$ for both groups), with the leg-leg PCI presenting the smallest values. The Q-PCI showed a significant difference in both groups from all other PCI values but the contralateral PCI and for the older adults group it was also significantly different from the ipsilateral PCI of the

right side (i.e., RaRl). PCI values for all other limbs' pairings did not differ from each other (p>0.06). To evaluate the consistency of the PCI values across the different limb pairings, we calculated Kendall's concordance coefficient. This analysis shows similar consistency between the two groups (young adults: W=0.46; older adults: W=0.45).

E. Supporting material for the *Results* section- *Subsection: Effect of arm swing amplitude on PCI values*

We correlated the arm swing amplitude with the PCI values of limbs' pairings that involve the relevant arm (i.e., right arm swing amplitude with PCI of limbs' pairing that involves the right arm). Here, we present the correlation for the young and the old adults separately. These correlations are presented in Table S3.

Table S3: Correlation between the arm swing amplitude and the correspondence PCI

Arms swing amplitude	Pair of limbs	YA correlation	OA correlation
Right hand	Arms	-0.51**	-0.5*
	RaRl	-0.49*	-0.34
	RaLl	-0.41*	-0.26
Left hand	Arms	-0.43*	-0.41*
	LaLl	-0.40*	-0.26
	LaRl	-0.46*	-0.35

YA: young adults; OA: old adults; Spearman correlation. *p<0.05; **p<0.0001

Supplementary material for:
A new measure for quantifying four-limb coordination of human gait based on mobility sensors

References for supplementary material:

1. Benady, A.; Zadik, S.; Ben-Gal, O.; Cano Porras, D.; Wenkert, A.; Gilaie-Dotan, S.; Plotnik, M. Vision Affects Gait Speed but Not Patterns of Muscle Activation During Inclined Walking—A Virtual Reality Study. *Front. Bioeng. Biotechnol.* **2021**, *9*, 632594, doi:10.3389/fbioe.2021.632594.
2. Benady, A.; Zadik, S.; Zeilig, G.; Gilaie-Dotan, S.; Plotnik, M. Gait Speed Modulations Are Proportional to Grades of Virtual Visual Slopes-A Virtual Reality Study. *Front. Neurol.* **2021**, *12*, 615242, doi:10.3389/fneur.2021.615242.
3. Cano Porras, D.; Zeilig, G.; Doniger, G.M.; Bahat, Y.; Inzelberg, R.; Plotnik, M. Seeing Gravity: Gait Adaptations to Visual and Physical Inclines − A Virtual Reality Study. *Front. Neurosci.* **2020**, *13*, 1308, doi:10.3389/fnins.2019.01308.
4. Rosenblum, U.; Kribus-Shmiel, L.; Zeilig, G.; Bahat, Y.; Kimel-Naor, S.; Melzer, I.; Plotnik, M. Novel Methodology for Assessing Total Recovery Time in Response to Unexpected Perturbations While Walking. *PLOS ONE* **2020**, *15*, e0233510, doi:10.1371/journal.pone.0233510.
5. Rosenblum, U.; Melzer, I.; Zeilig, G.; Plotnik, M. Muscle Activation Profile Is Modulated by Unexpected Balance Loss in Walking. *Gait Posture* **2022**, *93*, 64–72, doi:10.1016/j.gaitpost.2022.01.013.
6. Plotnik, M.; Arad, E.; Grinberg, A.; Salomon, M.; Bahat, Y.; Hassin-Baer, S.; Zeilig, G. Differential Gait Adaptation Patterns in Parkinson′s Disease - a Split Belt Treadmill Pilot Study. *BMC Neurol.* **2023**, *23*, 279, doi:10.1186/s12883-023-03321-4.
7. Heimler, B.; Koren, O.; Inzelberg, R.; Rosenblum, U.; Hassin-Baer, S.; Zeilig, G.; Bartsch, R.P.; Plotnik, M. Heart-Rate Variability as a New Marker for Freezing Predisposition in Parkinson′s Disease. *Parkinsonism Relat. Disord.* **2023**, *113*, 105476, doi:10.1016/j.parkreldis.2023.105476.
